# Reperfused human umbilical cords: hydrodynamic and hemolytic analysis of the *ex vivo* model for umbilical cord ECMO cannulation

**DOI:** 10.3389/fbioe.2026.1860486

**Published:** 2026-09-08

**Authors:** Jan Heyer, Lydia G. Jeremiah, Line Eichmann, Ritzy Sydney Melzer, Thorsten Orlikowsky, Mark Schoberer, Sebastian V. Jansen

**Affiliations:** 1 Cardiovascular Engineering, Applied Medical Engineering, Medical Faculty, RWTH Aachen University, Aachen, Germany; 2 Pediatric Clinic, Neonatology Section, Medical Faculty, RWTH Aachen University and University Hospital, Aachen, Germany

**Keywords:** artificial placenta, ECMO cannulation, extremely preterm infants, fetal blood, human umbilical cord

## Abstract

**Background:**

A new type of umbilical vascular access routes is needed for the extracorporeal blood circuits, which are part of artificial placenta treatment. So far, devices and techniques for this purpose have been developed and tested only in large animal models or simulators. We have previously reported a test setup for the development and training of umbilical vessel cannulation on reperfused human umbilical cords. It allows the simulation of the insertion of large-bore cannulae into the perfused umbilical arteries and vein, as it is planned to be performed in an ex utero intra partum procedure. We present an evolved version of the test stand with reduced priming volume, resistance and hemolysis.

**Methods:**

Standardization and optimization of cannulae and tubing reduced flow resistance and priming volume. Hematocrit was adjusted to 0.3. Fourteen umbilical cords were harvested, prepared, visually analyzed, and used to validate the setup. Seven circuits were primed with autologous placental blood at a hematocrit of 30%, and seven were primed with saline solution. A volume flow of 70 mL/min was targeted, and pressures and hemolysis were measured.

**Results:**

Differences in vessel structure and coiling were identified. The measured pressure drops were in a physiological range. There was no significant hemolysis caused by the cannulas or the umbilical cord compared to the tubing, reservoir, and blood pump.

**Conclusion:**

The test stand for reperfused human umbilical cord specimens primed with autologous placental blood or saline solution can provide a biosimilar model for medical therapies with low ethical concerns and patient risk.

## Introduction

About 13.4 million preterm infants were born worldwide in 2020 (Ohuma et al., 2023). Preterm birth is the leading cause of death among children under 5 years of age (Perin et al., 2022). Extremely preterm infants (EPI) are born before 28 weeks of gestational age (GA) and are the most vulnerable and immature patients with the highest mortality rates among preterm infants (Blencowe et al., 2013; Dougherty et al., 2022). Although mortality has decreased with progress in neonatal intensive care, mechanical ventilation (MV) used to assist with pulmonary gas exchange can cause lifelong organ damage, particularly to the immature lung (Regin et al., 2022; Hurskainen et al., 2021).

Artificial Amnion and Placenta technologies (AAPT) aim to enable organ maturation under physiological functional conditions which resemble pregnancy. The goal of this approach is to reduce the therapy-related morbidity of neonatal intensive care. In recent years, several research groups have developed promising approaches in artificial amnion and placenta technologies (AAPT) proving survival and growth of preterm lambs (Hornick et al., 2016; Partridge et al., 2017; van et al., 2025; Miura et al., 2016) and piglets (Charest-Pekeski et al., 2021). The gas exchange within the AAPT takes place in a dedicated oxygenator, connected through extracorporeal membrane oxygenation (ECMO) cannulas to the umbilical vessels (Miura et al., 2016; Charest-Pekeski et al., 2021; Arens et al., 2011; Hornick et al., 2018). These ECMO cannulas must resist dislocation and require a tight fit into the umbilical vessel, a low flow resistance, and non-hemolytic behavior. ECMO cannulation techniques of the umbilical cord have also been tested in lamb and piglet models (Hornick et al., 2018; Usuda et al., 2020). Owing to substantial differences in vessel count, vascular diameter, and tissue consistency between lamb and piglet umbilical cords and the human umbilical cord (UC), the results of animal trials can only be translated to human applications to a limited extent (Barrios, 2018; Randall, 1989; Jordan, 1919). We developed an *ex vivo* human umbilical cord test setup to simulate and test large-bore umbilical vessel cannulation of reperfused human umbilical cords. This technique is described by Hoyos et al. (2025).

The presented setup, however, did not fully represent physiological hydrodynamic properties of an extremely preterm infant of 24 weeks: While a target flow of 70 mL/min was achieved, the resulting pressure drops were unphysiologically high with 53.2 ± 32.7 mmHg along the umbilical arteries and 62.8 ± 23.4 mmHg along the umbilical vein. In addition, hematocrit (Hct) and viscosity of the utilized blood were not adjusted to standardized values.

The hemolysis produced within the previous circuit was not analyzed either. But hemolysis is one of the major complications in neonatal ECMO (Extracorporeal Life Support Organization, 2024), which could also be true for AAPT: The cannulation within AAPT with its narrow dimensions presents a region of high shear stress and hence high risk of hemolysis. While standard *in vitro* hemolysis testing is performed using either animal or human adult blood, neonatal blood markedly differs in its sensitivity towards blood damage as shown by Heyer et al. (2025). Therefore hemolysis should be investigated und tested for every component of an AAPT circuit.

Within this study, we present a progression of the *ex vivo* human umbilical cord test stand for AAPT large-bore cannulation. We report hydrodynamic data on the perfusion of human umbilical cords in this test stand, together with test stand-related hemolysis data. Results show improved physiological hydrodynamic characteristics, pressure drop, and connected volume flow. In addition, the test standard was developed with only commercially available components, enabling testing across different institutions human neonatal blood.

## Methods

### Ethics

Human umbilical cords and were used and reperfused with autologous blood within the *ex-vivo* model. Written maternal consent was obtained in all cases. The umbilical cords and blood were collected from the RWTH centralized biomaterial bank (cBMB) directly upon delivery via caesarean section. The ethics committee of the medical faculty of RWTH Aachen University (EK 206/09) specifically approved this procedure for the corresponding project. Data from the mother, the child and the harvested materials were pseudonymized by the cBMB. The lookup table is only accessible to the project physicians and the cBMB.

### Blood and umbilical cord harvesting

The methodology of blood and umbilical cord harvesting is described by Hoyos et al. (2025). Briefly, placental blood was drained from the placenta and UC aiming for a volume of more than 50 mL for priming of the *ex-vivo* test stand. Subsequently, the UC was cut from the placenta. Exclusion criteria for UC and blood harvesting were: minor legal age of the mother, refusal of maternal consent, maternal or fetal infectious diseases, vaginal births, UC length under 15 cm, and a UC with fewer than three patent vessels.

After harvesting, both ends of the umbilical cord segment were terminally cannulated with 50 mm 8 Fr Ultimum TM introducer sheaths (Abbott Medical, Plymouth, USA) in each vessel. 8 Fr (inner diameter of 1.67 mm) DCT Nelaton Catheters (Servoprax GmbH, Wesel, Germany), shortened to a length of 50 mm, were inserted into the introducer sheaths. Cable ties padded with foam tape were hand-fastened around each end of the UC to fixate terminal cannulation for transfer from the hospital to the lab.

At the lab, the cable ties were finally tightened (68 N cut-off force), and the umbilical cord was primed with 0.9% saline solution (NaCl 0.9% B. Braun Melsungen AG, 34209 Melsungen, Germany). The cannulated cord was weighed before and after priming to assess total priming volume. The harvested blood was filtered and filled into a 150 mL 2-port PVC blood bag (Qosina Corp., Ronkonkoma, NY 11779, USA). Hct was determined by microhematocrit centrifugation. The blood was diluted with 0.9% saline solution to adjust to a Hct of 30% ± 1%, resulting in a minimum of 80 mL blood for testing.

### *Ex vivo* umbilical cord testing setup

**FIGURE 1:**
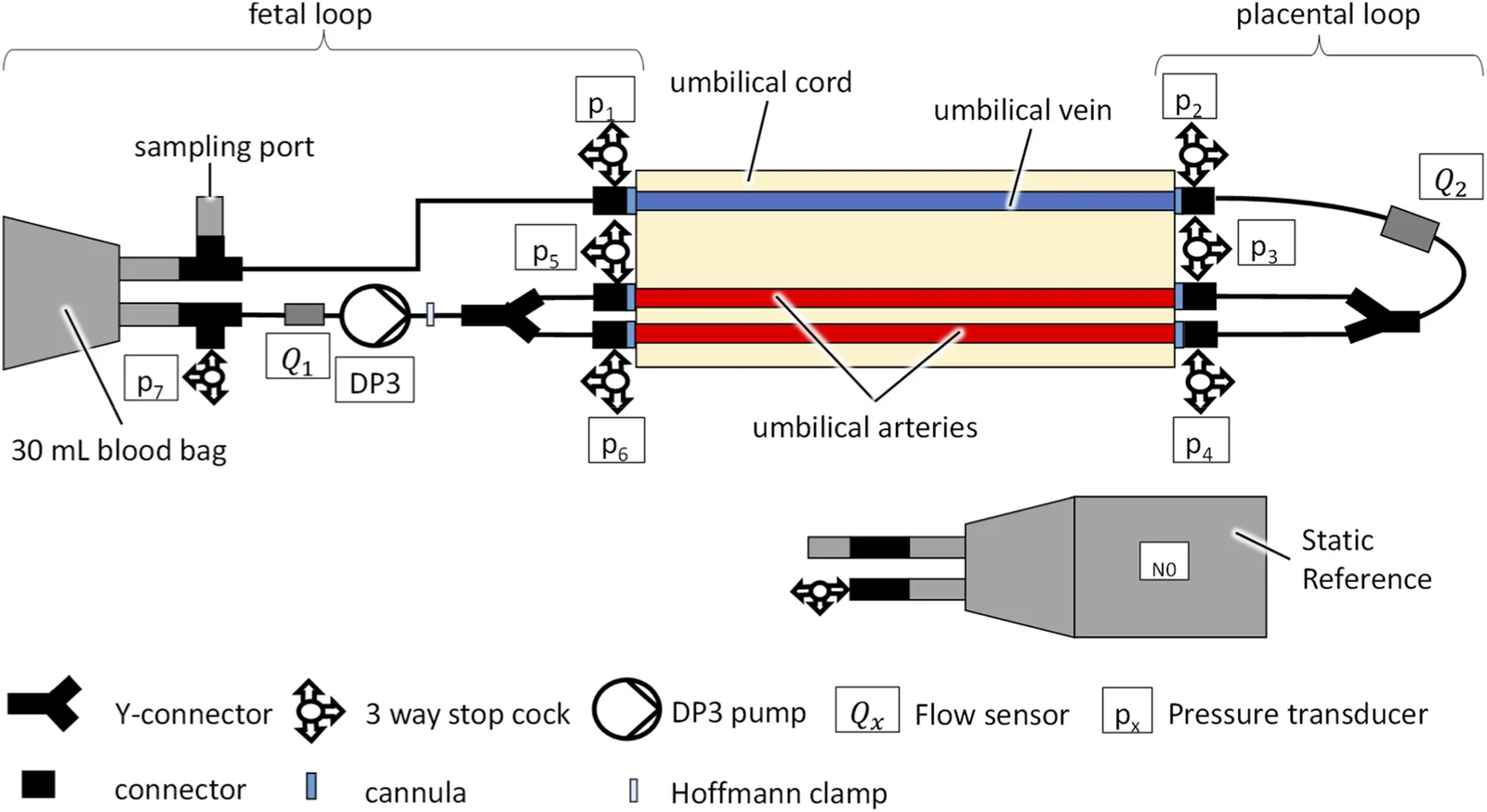
Test circuit of the new, modified *ex vivo* human umbilical cord setup, based on the works of Hoyos-Banchon et al.

**FIGURE 2:**
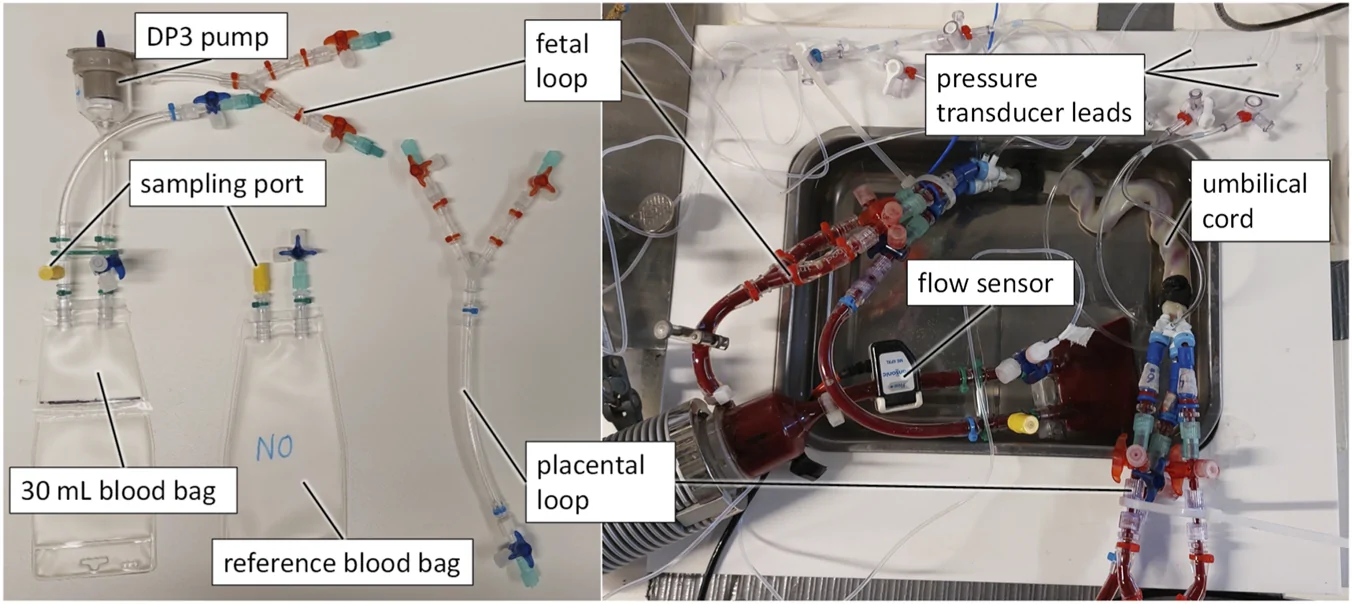
Photos of empty test circuit (left) and circuit during experiment (right).

The test setup (Figure 1) consisted of a test circuit and a static reference. The test circuit (Figure 2) was adapted from the one presented by Hoyos et al. (2025) and further standardized and optimized. The circuit consisted of a fetal and a placental loop. The fetal loop represents a simplification of the fetus’ cardiac system, whereas the placental loop acts as an arteriovenous bypass by connecting both arteries to the vein. Different from Hoyos-Banchon et al.’s setup, a 1/4 inch Deltastream© DP3 pump was used, and a 2-port PVC blood bag, adjusted to a volume of 30 mL (Qosina Corp., Ronkonkoma, NY 11779, USA), replaced the tailor-made reservoir. A blood sampling port (B. Braun Melsungen AG, 34212 Melsungen, Germany) was added to the fetal loop to enable hemolysis testing. A Hoffmann clamp was placed downstream of the blood pump to adjust the flow rate. Total circuit volume was 70 mL plus the additional, variable volume of the umbilical cord vessels (5.9 mL ± 3.6 mL).

Prior to the experiment, fetal and placental loops were rinsed using 0.9% saline solution, before the UC was connected and the whole setup was primed with the adjusted human autologous blood (displacing the saline solution in the previously primed UC). After priming, the whole circuit was checked for air inclusion and vented if necessary. During the experiment, the umbilical cord and the reservoir were submerged in a basin containing 1.5 L Ringer solution. The temperature in the basin was controlled using a temperature-controlled water bath to maintain a temperature of 38 °C–39 °C. This temperature was determined in previous experiments to result in a blood temperature of 37 °C ± 0.3 in the flow loop. Volume flow was measured by connecting two flow sensors (Getinge Deutschland GmbH, 76437 Rastatt, Germany) to the fetal and placental loop. Pressures were measured up- and downstream of each umbilical vessel (p_1_ to p_6_) and in the reservoir (p_7_) using Xtrans pressure transducers (Codan pvd Critical Care GmbH, 85661 Forstinning, Germany). The static reference (N0) consisted of a blood bag (Qosina, Ronkonkoma United States) and was placed in a separate water bath at a temperature of 37 °C. The static reference determines the autologous hemolysis of the blood and serves as a baseline for hemolysis testing.

When the amount of harvested blood was not sufficient to perform the experiment, it was conducted using 0.9% saline solution to determine the hydrodynamic behavior of the UC with saline solution. Hemolysis testing was not performed in this cases.

### Visual assessment of umbilical cord geometry

The length and circumference of the umbilical cord were measured during the initial preparation of the umbilical cord. The umbilical cord outer diameter (D_UC_) was approximated from the mean umbilical cord circumference (C_UC_), measured at three different locations, using D_UC_ = C_UC_/π. The Umbilical Coiling Index (UCI) was determined by the ratio of umbilical coils to the umbilical cord length in centimetres (Wilke et al., 2021). Photos were taken before, during and after the experiment. The increase in D_UC_ was optically determined by evaluation of the photos. In addition, UC vessels were checked for anatomical irregularities.

### Hydrodynamic testing

The hydrodynamic experiments aimed to measure the flow-pressure relationship of the new standardized *ex vivo* testing setup. Umbilical vessels show a high inter-individual variability regarding shape, course, diameter and elasticity (Pergialiotis et al., 2020; Proctor et al., 2013; Wang et al., 2022). They are almost completely constricted at the beginning of the experiment; the hydrodynamic behavior after reperfusion is not analytically predictable and hence was measured here.

The experiment was divided into two phases. Perfusion started with a ramp-up phase to slowly counteract the spasm of the UC. Over 60 min, the flow was increased in 10 mL/min increments from 10 mL/min to 70 mL/min every 10 min. Rotational velocity of the pump was set to defined values for each flow rate, and the Hoffmann clamp was used to fine-tune the flow rate, compensating for individual anatomical differences in the UC. In the cases where the test circuit was filled only with saline solution, the experiment was terminated after the ramp-up phase.

In the other cases, the ramp-up phase was followed by a constant flow phase to investigate hydrodynamics and hemolysis over time. During this phase, a flow rate of 70 mL/min ± 5 mL/min (RPM = 4300 1/min) was set for a duration of 3 h. At the end of the constant flow phase, 5 mL of blood were extracted from the test circuit for viscosity measurement. The pressure drop over the umbilical cord plus placental loop was calculated as the pressure difference of the mean of pressures p_5_, p_6_ and p_1,_ according to Equation 1:

$$\Delta p=\frac{p_{5}+p_{6}}{2}-p_{1}$$ (1)

### Hemolysis testing

During the constant flow test phase, blood samples for hemolysis testing were taken from the test circuit and the static reference (N0) at 0, 30, 60, 120, and 180 min. Samples were taken directly from the blood flow via the sampling port with a 1 mL syringe using a 23 G cannula. Blood count was measured using a hematology analyzer (ProCyte Dx, IDEXX, Kornwestheim, Germany). Hemolysis was then assessed by the concentration of free-plasma hemoglobin (fHb) according to the cyanohemoglobin method standardized in DIN 58931 2026. The normalized index of hemolysis (NIH) was calculated from the fHb according to Equation 2:

$$NIH=\frac{\Delta fHb\cdot V\cdot \frac{100-Hct}{100}}{Q\cdot \Delta T\cdot 1000}\;\left[\frac{g}{100\;L}\right]$$ (2)

ΔfHb: difference in free plasma hemoglobin in the considered sampling interval in mg/dL
V: blood volume in the testing circuit in mL
Hct: Hematocrit in %
Q: blood flow rate in L/min
ΔT: time interval length in min

NIH data were tested for normal distribution using the Shapiro-Wilk test. A 2-sample t-test was performed to compare the NIH data from the present study with the data from the study by Heyer et al. (Heyer et al., 2025). In addition, Pearson correlations of NIH with umbilical cord parameters (GA, birthweight (BW) of the child, visually assessed geometry of UC) were calculated.

## Results

Between July 2024 and September 2024 14 umbilical cords were harvested and successfully connected to the test circuit. GA ranged from 37+0 to 39+3 weeks. Birth weight (BW) ranged from 2,665 g to 4,270 g. Among the 14 experiments, seven were performed using autologous blood (“blood group”), and seven using saline solution (“NaCl group”). The target flow of 70 mL/min was successfully established in all cases. The mean dynamic viscosity of the 30% Hct adjusted blood was determined to 2.2 ± 0.3 mPas in the blood group, and 1.07 mPas (Ko et al., 2022) in the NaCl group. Two of the seven blood group experiments were terminated during the constant flow testing phase due to the formation of a hematoma in the umbilical cord.

### Visual assessment of umbilical cord geometry

**FIGURE 3:**
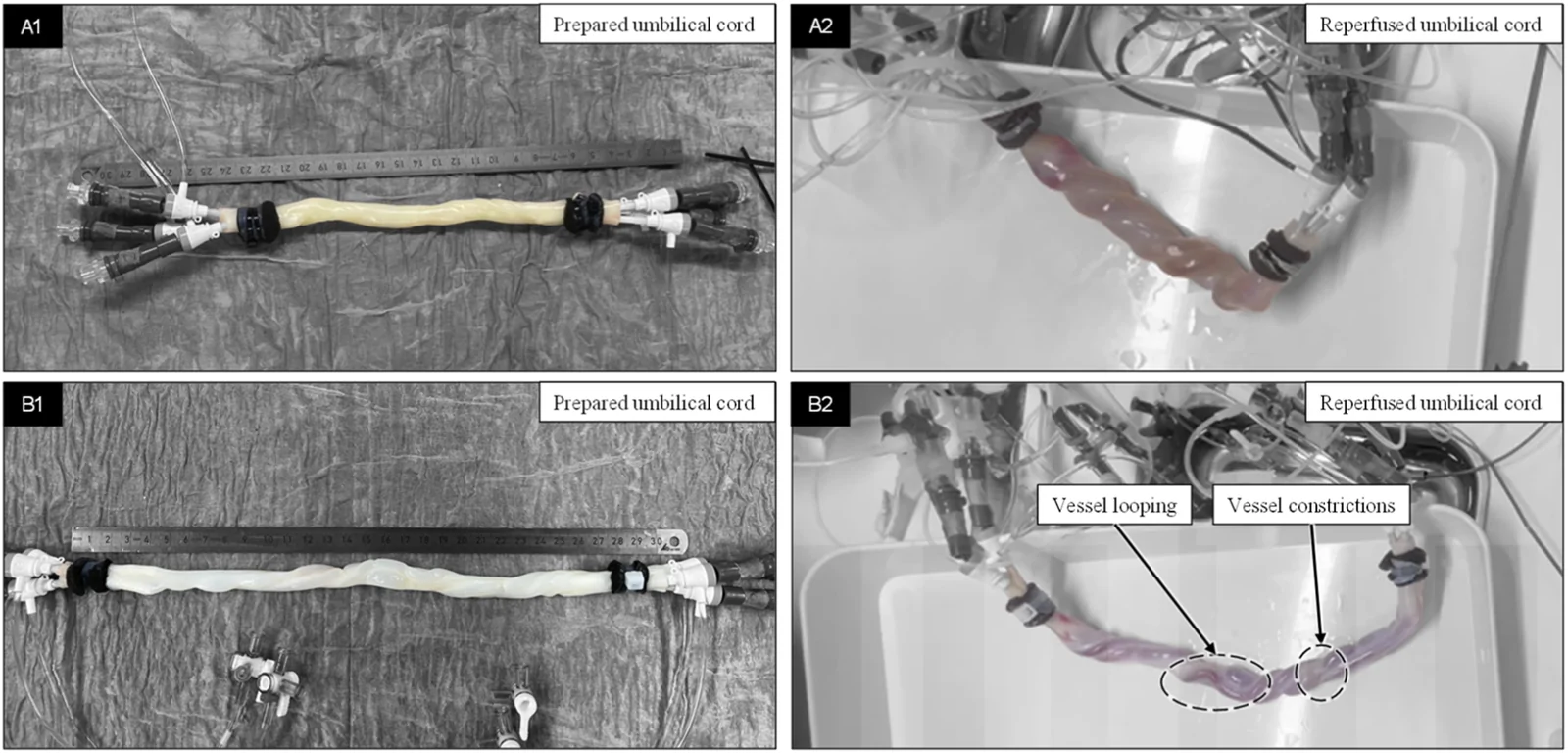
Visual assessment of two umbilical cords **(A,B)** before (left) and after (right) reperfusion: **(A1,A2)** helical shape of umbilical arteries around the vein; **(B1,B2)** irregular looping shape of the arteries around the vein.

The harvested umbilical cord lengths ranged from 15.0 cm to 32.7 cm (n = 14 UCs). The coiling indexes were calculated to 0.049 to 0.150 coils per centimeter. Mean outer umbilical cord diameters (D_UC_) ranged from 8.6 mm to 17.7 mm (mean 12.0 ± 2.4 mm) and from 13.1 mm to 21 mm (mean 15.7 ± 2.7 mm) before and after the experiment, respectively. This corresponds to an average increase of D_UC_ during reperfusion by a factor of 1.34 ± 0.25. Five UCs presented with geometric irregularities, including vessel constrictions and looping. Figure 3 shows two exemplary umbilical cords, without (A) and with (B) irregularities, before and after reperfusion.

### Hydrodynamic testing results

**FIGURE 4:**
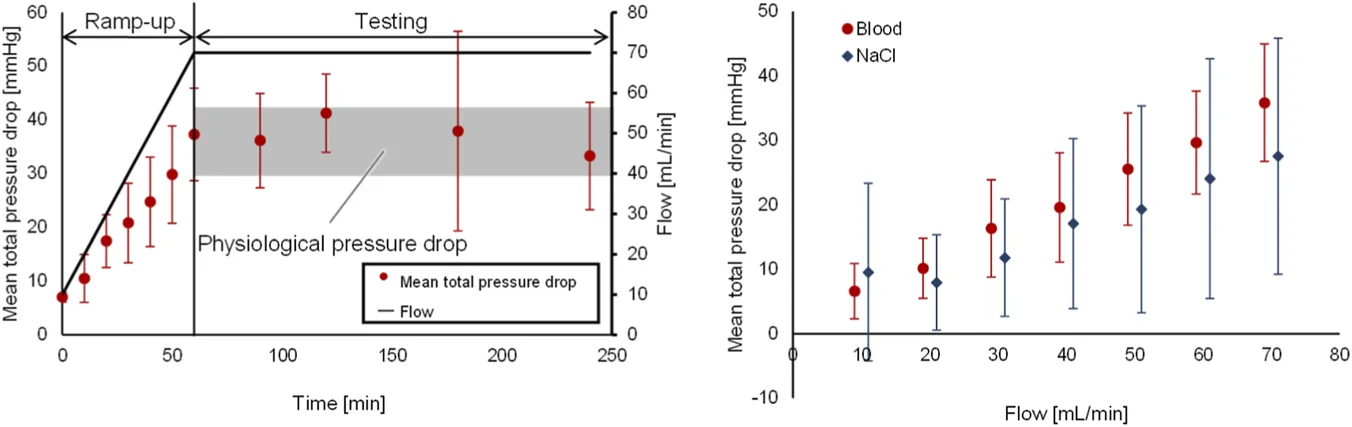
Mean total pressure drop and volume flow plotted against time for the hole experiment (right); mean pressure drop over umbilical cord plus placental loop during the ramp-up phase with blood and NaCl solution plotted against flow (left).

Figure 4 (right) shows the mean total pressure drop and flow for the blood group (n = 7) over the umbilical cord plus placental loop in the ramp-up and constant flow testing phase over time. A list of the mean resulting pressures during the testing phase is shown in Supplementary Material S1. Over the course of the experiment, the total pressure drop increases linearly with flow during ramp-up, whereas during the constant-flow phase, it decreases slightly over time. Figure 4 (right) gives a detailed excerpt of the ramp-up phase with total pressure drop for both fluid groups (blood and NaCl). In general, both groups show a linear increase of pressure drop with increasing flow, with the NaCl group (n = 7) presenting a lower gradient, as expected due to the lower viscosity.

**FIGURE 5:**
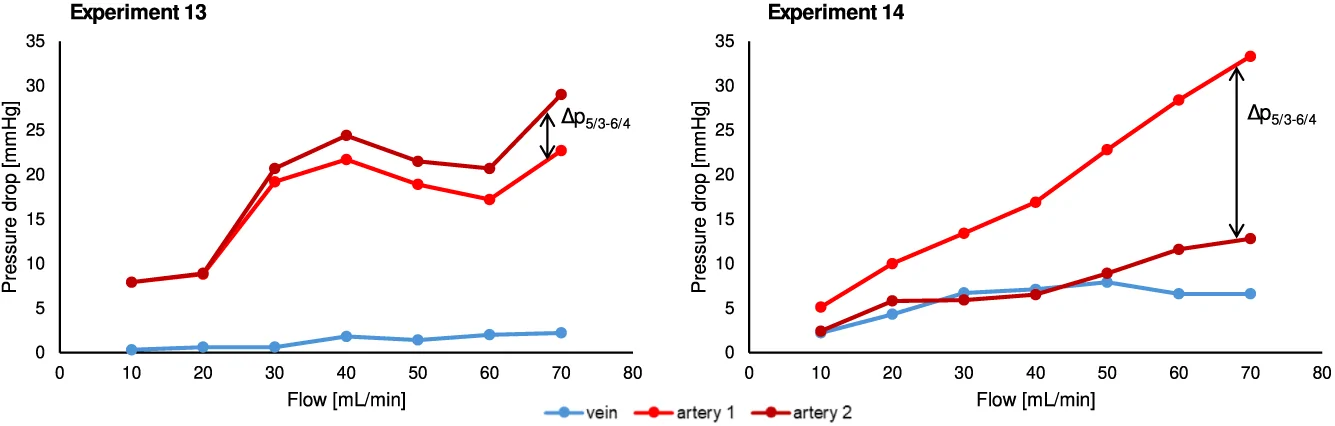
Individual hydrodynamic behavior of two exemplary umbilical cords UC 13 (left) and UC 14 (right) during the ramp-up phase, with the pressures of each umbilical artery (p_5/3_ and p_6/4_) and the umbilical vessel (p_2/1_).

Standard deviation of both groups is relatively high, demonstrating the highly individual hydrodynamics of the tested umbilical cords. This is largely attributed to individual geometric variations and potential spasms during ramp-up. Figure 5 gives two examples of such individual behaviour with UC 13 presenting a non-linear pressure drop with increasing flow (left), and UC 14 showcasing a different pressure drop over each artery (right). In general, the pressure drops of the veins were lower compared to the arteries, but also highly individual with the vein pressure drop in UC 14 being twice as high as in UC 13 for example. The shown Δp_5/3–6/4_ describes the difference in pressure drop among the umbilical arteries.

In two of the seven UC in the blood group, a small hematoma was detected near the terminal cannulation. However, no impact on the hydrodynamics that would exceed the variation due to the UC’s individuality was observed.

### Hemolysis testing results

**FIGURE 6:**
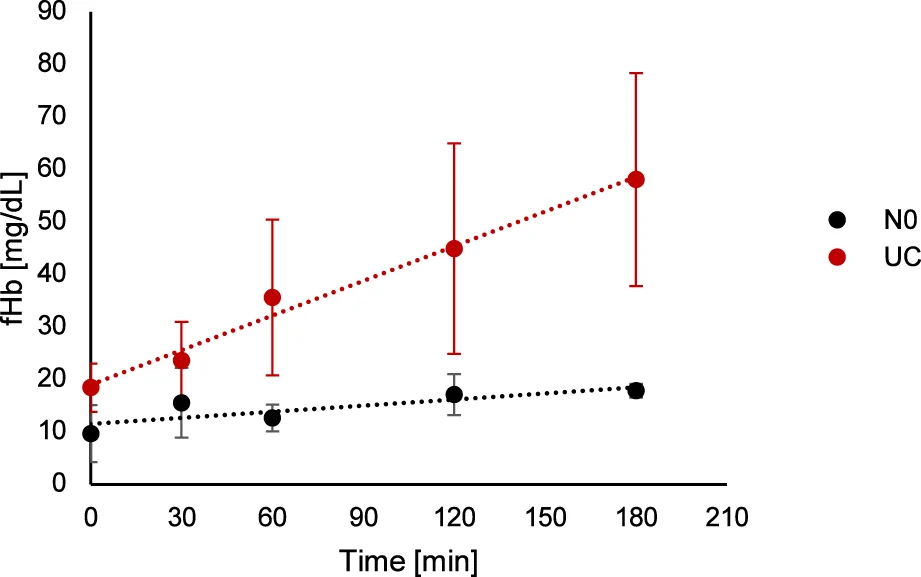
Mean fHb in the static reference samples (N0) and testing circuit samples (UC) plotted against time during the constant flow phase.

Of the n = 7 umbilical cords in the blood group, two were excluded from the hemolysis testing due to the formation of a hematoma in the UC. Figure 6 shows the mean of the measured plasma-free hemoglobin (fHb) of the test circuit (n = 5), and the static reference samples (N0) during the constant flow phase. Both groups present an increasing fHb over time.

**FIGURE 7:**
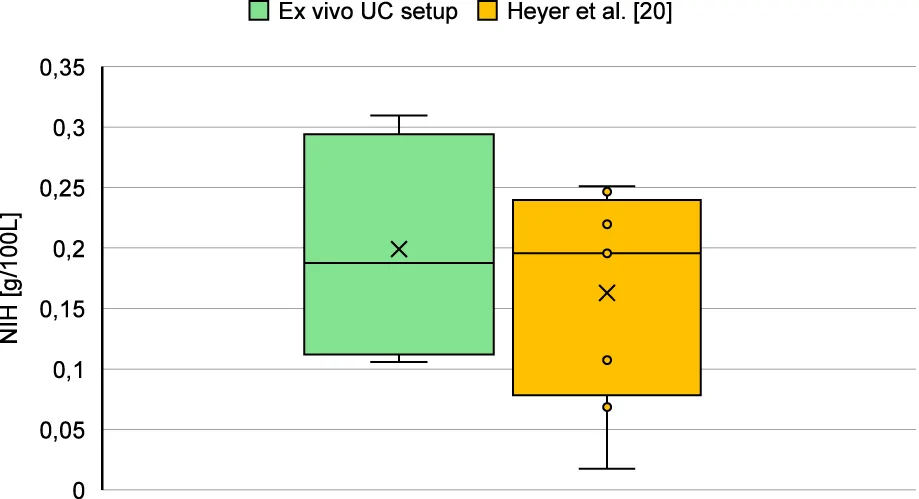
NIH-Data in the present study (UC) and the previous study performed by Heyer et al. (2025).

NIH was calculated from fHb and found to be normally distributed according to the Shapiro-Wilk test, with an average of 0.199 ± 0.091 g/100L (mean ± SD). Figure 7 shows the herein measured NIH data (UC) in comparison to the results of a previous study (Heyer et al., 2025), which measured hemolysis within the test circuit alone (not including the umbilical cord and its cannulation). It shows a similar range with no significant difference (p = 0.465) found between both data sets, indicating that the umbilical cord and its cannulation is not a major contributor to the overall hemolysis in the test setup.

Pearson correlation coefficients r for NIH with GA, BW, L_UC_, D_UC_, and UCI are given in Table 1. All r values are below ± 0.30, suggesting only a weak to no correlation.

**TABLE 1:** Pearson correlation coefficients r for correlation with NIH.

Correlation coefficient	GA [weeks]	BW [g]	L_UC_ [cm]	D_UC_ [mm]	UCI [coils/cm]
r	−0.268	0.146	0.245	−0.210	0.036

## Discussion

The study presented here demonstrates the successful optimization and hemolytic and hydrodynamic characterization of the *ex vivo* test stand for reperfused human umbilical cords. The original test stand, as presented in Hoyos-Banchon et al., was limited by unphysiological pressures and non-standardized circuit parts. By the use of terminal cannulas, the flow resistance within the circuit decreased. A physiological pressure drop of 30–40 mmHg of the UC and placental loop was achieved. The vessel pressures were calculated from measurements before and after the umbilical cord, with the theoretical pressure loss of the cannulas calculated by the Hagen-Poiseuille law. The arteries with a mean value of 36.06 mmHg had a physiological pressure and the umbilical vein with a mean of 21.78 mmHg had a slightly increased physiological pressure (cf. Supplementary Material S1). Therefore, it is possible to cannulate a human umbilical cord with physiological pressures within our test stand. The slightly increased pressure within the vein could be used as a conservative assumption for functional cannulation.

Furthermore, the introduction of catheters into the introducer sheaths increased the stability of the cannulation and prevented ‘crushing’ of the cannulation with an increased flow resistance. However, the introducer sheaths were also likely to be the cause of the two hematomas found in umbilical cords, since these formed directly at the tips of the sheaths. Only commercially available parts were used for the test stand, so that the proposed setup and the herein found characteristics can serve as a benchmark for standardized umbilical cord testing across different research groups.

The hydrodynamic testing revealed, on average, a linear relationship between increasing flow and mean pressure drop during the ramp-up phase, despite complex anatomical geometries and potential spasm in reperfused umbilical cords. However, high standard deviations were also observed, which are linked to the highly individual characteristics of each umbilical cord. The generally observed decrease in total pressure drop (together with an increase in umbilical cord outer diameter) over time during the constant-flow phase is likely to result from further relaxation of umbilical cord spasm after prolonged reperfusion. It cannot be ruled out that the pressure drop would decrease further with longer reperfusion times.

With the NaCl group, a lower pressure drop was observed, as expected from the lower viscosity (1.07 mPas (Ko et al., 2022) vs. 2.2 mPas in the blood group). The results of the NaCl group show that the target volume flow can also be achieved with saline solution for reperfusion rather than autologous blood. Comparing the different pressure values relative to the viscosities of NaCl and blood could lead to additional effects depending on the priming fluid. If the viscosity doubles, the pressure differences should also double. We identified two potential effects that could explain the absence of high pressure when viscosity is doubled. First, the greater pressure variability observed in the NaCl group suggests that blood as a priming fluid and the corresponding access to blood gases may relieve vasospasm more effectively than NaCl, yielding a larger effective vessel lumen that would partially offset its higher viscosity. Second, the high anatomical and functional variability of the vessels, potential vessel loops and vascular contractions cause a high standard deviation in our experiments. Differences in compliance might alter damping effects within the cords. Future examinations ought to include a larger number of experiments to further clarify the interrelationship of priming fluid and blood pressure (cf. Figures 3, 5).

The measured fHb increased linearly in all experiments. A comparison of NIH values with the study of Heyer et al. 2025, where hemolysis of the circuit without UC and cannulation was assessed, showed no significant difference. Therefore, we assume that the umbilical cord and the terminal cannulation do not increase hemolysis within the circuit to a relevant extent. In addition, the absolute NIH value of the test stand can be categorized as mild according to Lou et al. (2014). This enables hemolysis testing of cannulas and AAPT components. No correlation between umbilical cord parameters and the NIH was identified. Hence, the highly individual anatomical differences of the n = 5 umbilical cords tested herein did not have an impact on hemolysis generation. The influence of donor-donor variability on the NIH seems to be low. However, this hypothesis needs to be tested in further experiments.

The harvested umbilical cords were derived from patients born after 36_+0_ weeks of GA, which is older than the targeted patient group for AAPT applications (24_+0_ GA). However, we assume that the properties of a 24_+0_ weeks of gestational age EPI could be mimicked with the harvested cords. The outer diameter of the tested umbilical cords was calculated to 12.0 mm ± 2.4 mm. Compared to *in utero* measurements of around 12 mm of fetuses in the 24 weeks of GA (Weissman et al., 1994; Raio et al., 1999). Hence, the tested umbilical cords are within a physiological range. Due to the linear viscoelastic behavior of the umbilical cord (Pennati, 2001), the vessel geometry is regulated by the tissue, dependent on the given hydrodynamics. Therefore, physiological diameters can be achieved with harvested umbilical cords from higher-gestational-age fetuses.

The *in vitro* evaluation of hemocompatibility, including coagulation, thrombus formation, and potential inflammatory responses, is a general challenge in blood testing, since there are no fully developed standardized models. In terms of AAPT, anticoagulation strategies are also under development, and one of the remaining challenges. Anticoagulation strategies depend on the intended outcome, the corresponding standards, the *in vitro* test system, or the animal model used in in vivo testing. In further study, the setup presented here could be characterized in terms of hemocompatibility. The anticoagulants used and their doses could be varied and analyzed for activated clotting time. These results could be taken to assess potential clot formation or behavior during cannulation, within cannulas, or within the umbilical cord in human tissue prior to *in vivo* experiments.

The study is limited by a small donor sample, which complicates statistical comparisons but is consistent with testing standards. The calculated umbilical cord diameter was assumed circular, which could slightly distort the true geometric result. The mean pressure drop within the test stand and the vessel pressures are within physiological ranges, but with venous pressures at the upper border of physiology. The test stand can be operated with a physiological mean pressure drop and a physiological vessel pressure. If needed, different pressure arrangements could be achieved by further adjusting the pressure with the Hoffman clamp. Looking at the individual pressure drops of the arterial branches, the resulting values are unequal due to loss coefficients while separating and combining the volume flow. In future experiments, the volume flow at the different branches needs to be additionally measured to fully characterize the vessel resistances.

## Conclusion

The study successfully optimized and standardized the *ex vivo* test setup for reperfused human umbilical cords. The test stand is reproducibly usable for standardized human umbilical cord cannulation, hemolysis estimation, physiological hydrodynamic testing, or other procedures with minimal ethical concerns and patient risk.

## Data Availability

The raw data supporting the conclusions of this article will be made available by the authors, without undue reservation.
