# Supplementary material for: Reperfused human umbilical cords: hydrodynamic and hemolytic analysis of the ex vivo model for umbilical cord ECMO cannulation
Source: Front Bioeng Biotechnol. 2026 Sep 8;14:1860486. doi: 10.3389/fbioe.2026.1860486 (PMC13593832; doi:10.3389/fbioe.2026.1860486)
Supplement: Supplementary file 1 [file DataSheet1.pdf]

Cord	Fluid	Flow in mL/min	All pressures are in mmHg										
			p1	p2	p3	p4	p5	p6	p7	mean p5 and p6	Placntal loop pressure drop	Mean arterial pressure	Mean vein pressure
1	Blood	70	8,84	12,4	23,3	27,5	31,7	32	-1,68	31,85	22,76	26,55	18
2			7,2	14,9	31,5	29,3	40,8	44,1	-2,5	42,45	34,5	37,15	26,2
3			8,14	18,2	28,62	40,52	52,24	43,04	-0,86	47,64	35,15	42,34	23,32
4			8,025	8,225	24,875	22,775	42,425	44,625	-2,7	43,525	35,8	38,225	19,575
5			8,05125	13,43125	27,07375	30,02375	41,79125	40,94125	-1,935	41,36625	32,0525	36,06625	21,77375
6	0,9 % NaCl Solution	70	3,56	24,02	33,16	33,12	36,7	36,68	-0,74	36,69	32,48	31,39	27,86
7			4,74	5,62	16,02	15,28	17,2	17,6	-2,62	17,4	12,66	12,1	10,72
8			5,06	5,42	15,92	13,36	27,22	23,82	-2,76	25,52	20,91	20,22	10,62
9			1,175	29,375	40,875	35,25	71,475	78,375	-0,825	74,925	70,1	69,625	35,575
10			4,82	7,64	14,44	14,54	17,82	18,22	-1,98	18,02	14,6	12,72	9,14
11			2,32	3,78	9,78	13,88	24,2	19,76	-3,14	21,98	18,41	16,68	4,48
12			4,4	4,5	11	10,3	36,7	34	-2	35,35	29,55	30,05	5,7
